# An appraisal of theoretical approaches to examining behaviours in relation to Human Papillomavirus (HPV) vaccination of young women

**DOI:** 10.1016/j.ypmed.2015.08.004

**Published:** 2015-12

**Authors:** Harriet Batista Ferrer, Suzanne Audrey, Caroline Trotter, Matthew Hickman

**Affiliations:** a School of Social and Community Medicine, University of Bristol, Bristol BS8 2PS, United Kingdom; b Department of Veterinary Medicine, University of Cambridge, Cambridge, CB3 0ES, United Kingdom

**Keywords:** HPV, Human Papillomavirus, UK, United Kingdom, USA, United States of America, Models Theoretical, Adolescent, HPV vaccines

## Abstract

**Background:**

Interventions to increase uptake of Human Papillomavirus (HPV) vaccination by young women may be more effective if they are underpinned by an appropriate theoretical model or framework. The aims of this review were: to describe the theoretical models or frameworks used to explain behaviours in relation to HPV vaccination of young women, and: to consider the appropriateness of the theoretical models or frameworks used for informing the development of interventions to increase uptake.

**Methods:**

Primary studies were identified through a comprehensive search of databases from inception to December 2013.

**Results:**

Thirty-four relevant studies were identified, of which 31 incorporated psychological health behaviour models or frameworks and three used socio-cultural models or theories. The primary studies used a variety of approaches to measure a diverse range of outcomes in relation to behaviours of professionals, parents, and young women. The majority appeared to use theory appropriately throughout. About half of the quantitative studies presented data in relation to goodness of fit tests and the proportion of the variability in the data.

**Conclusion:**

Due to diverse approaches and inconsistent findings across studies, the current contribution of theory to understanding and promoting HPV vaccination uptake is difficult to assess. Ecological frameworks encourage the integration of individual and social approaches by encouraging exploration of the intrapersonal, interpersonal, organisational, community and policy levels when examining public health issues. Given the small number of studies using such approach, combined with the importance of these factors in predicting behaviour, more research in this area is warranted.

## Highlights

• Interventions to increase HPV vaccine uptake should incorporate appropriate theory.
• Psychological health behaviour theories predominate in studies of HPV vaccination.
• The wider determinants of HPV vaccine uptake should be examined.
• Ecological approaches offer a framework to examine personal and social factors.

## Introduction

Globally, inequalities in the incidence of cervical cancer exist by geographic area (Arbyn et al., 2011, Shack et al., 2008), socioeconomic status (Shack et al., 2008, Parikh et al., 2003, Singh et al., 2004) and ethnicity (National Cancer Intelligence Network, 2009, Watson et al., 2008). Since licensure in 2006, many countries have introduced the Human Papillomavirus (HPV) vaccine into their national immunisation programmes for the primary prevention of cervical cancer. High coverage has the potential to reduce substantially cervical cancer incidence and mortality (Harper et al., 2004, Harper et al., 2006, Garland et al., 2007, Garland et al., 2009).

However, there is the potential to increase health inequalities if vaccine uptake is lower amongst less affluent and marginalised populations that may also have greater risk of developing cervical cancer. Evidence for differences in uptake has been shown. Globally, evidence from a systematic review and meta-analysis did not indicate strong evidence for associations by socioeconomic variables, but young women belonging to minority ethnic groups were less likely to receive the HPV vaccine (Fisher et al., 2013). Further in the United States of America (USA), which delivers the HPV vaccination programme in the healthcare setting, young women without healthcare insurance coverage were less likely to be vaccinated (Fisher et al., 2013).

In relation to the United Kingdom (UK) routine school-based programme, studies have indicated a lack of association between initiation of the HPV vaccination course and area-level measures of deprivation (Sinka et al., 2013, Spencer et al., 2014, Fisher et al., 2014). However, lower uptake by minority ethnic young women has been reported (Spencer et al., 2014, Fisher et al., 2014, Bowyer et al., 2014). In the catch-up programme, a higher level of deprivation is associated with a lower odd of initiation and completion of the HPV vaccination course (Sinka et al., 2013, Spencer et al., 2014, Beer et al., 2014). Despite free access within the Danish healthcare-based programme, inequality by ethnicity and socioeconomic factors has been shown (Slattelid Schreiber et al., 2015). Further, in the Norwegian school based programme, lower rates of initiation were reported for young women with mothers belonging to the lowest income group (Hansen et al., 2015).

The UK Medical Research Council Framework argues that complex interventions, such as an intervention to increase HPV vaccination uptake, should be developed and underpinned by appropriate theory which captures the likely process of behaviour change (Craig et al., 2008, Campbell et al., 2000). There is growing evidence that interventions developed with an explicit theoretical foundation are more effective and more likely to induce positive behaviour change (Glanz et al., 1990, Michie et al., 2008). Therefore, the aims of this literature review are: to provide an overview of theoretical models and frameworks that have been used in published research to explain behaviours in relation to HPV vaccination of young women, and: to consider the appropriateness of the theoretical models or frameworks for informing the development of interventions to increase uptake.

## Materials and methods

Methods to identify relevant primary studies were based on those previously used by the study authors for systematic reviews in the field of HPV vaccination (Fisher et al., 2013, Batista Ferrer et al., 2014). There are differences between ‘models’, ‘frameworks’, and ‘theories’. Theories tend to be specific, with concepts which are amenable to hypothesis testing whereas models tend to be more prescriptive, specific and with a narrower scope. Conceptual frameworks are usually descriptive, showing relevant concepts and how they relate to each other (Ilott et al., n.a). Throughout the manuscript, we use the terminology ‘models’, ‘frameworks’ and ‘theories’ consistent with their use by the authors of included studies.

### Search strategy

To identify the relevant literature in relation to the HPV vaccine and theoretical models and frameworks, a search strategy previously used elsewhere (Fisher et al., 2013, Batista Ferrer et al., 2014) was adapted which comprised the following combination of text words (searching the title and abstract) and Medical Subject Heading (MeSH) terms: [‘papillomavirus’ or ‘wart virus’], [‘vaccination’ or ‘immunisation’ or ‘immunization programmes’ or ‘wart virus vaccines’] and [‘theory’ or ‘theoretical model’]. The following databases were searched from inception to 5th December 2013: CINAHL; Embase; Medline; PsycINFO; and ISI Web of Science & ISI Proceedings. All abstracts were saved using Endnote X3 reference manager software.

After duplicates were removed, all titles and abstracts of identified studies were assessed by one author (HB-F) to consider their relevance for inclusion. Two reviewers independently reviewed potentially relevant full texts (HB-F, JC). Disagreements were resolved by discussion.

### Eligibility criteria

Studies were eligible for inclusion if a theoretical model or framework was explicitly used in the study design and the study reported findings to explain behaviours in relation to the HPV vaccination of young women aged nine to 18 years old. Any study design, including qualitative and quantitative approaches, was eligible. No restrictions were imposed on the basis of publication date or language. Potentially relevant conference abstracts or dissertations were checked to see if a full paper had been published in a journal. Reference lists and citation lists of primary studies and relevant systematic reviews were hand searched for additional references.

### Data extraction

Study characteristics (authors, year of publication, study aims, study design, participants) and details related to use of theoretical model of framework (theoretical model, analytical approach, overall study findings, data to evaluate the use of theory) were extracted and entered into an excel spread sheet by one study author (HB-F) and doubled checked by another (SA).

### Assessment of use of theoretical model or framework in primary studies

Presently, there is no standardised method or consensus for assessing use of theory within studies which can vary within different research disciplines. To assess the level of use of theory by the primary studies, the study was assessed as either: (i) partially applied: authors locate their study within a particular theory but then appear to abandon efforts to link, apply, or interpret their findings in relation to that context, or; (ii) consistently applied: the theory guides and directs the various phases of the research process and can be tracked throughout the primary study (Bradbury-Jones et al., 2014). This was quantified by assessing whether the authors justified their use of theory within the introduction or methods, presented their results in relation to the theory and whether they made reference to the theory within the discussion.

This assessment was adapted from a five-point typology proposed by Bradbury-Jones et al. on the levels of theoretical visibility which includes ‘implied’, ‘seemingly absent’, ‘partially applied’, ‘retrospectively applied’, and ‘consistently applied’ (Bradbury-Jones et al., 2014). Primary studies were eligible for inclusion to the present study if a theoretical model or framework was explicitly used in the study design. Therefore, the categories ‘implied’ and ‘seemingly absent’ were not applicable to the present study and were not used. As discussed by Bradbury-Jones et al., studies which retrospectively apply theory are almost impossible to detect as the study authors often do not make this explicit (Bradbury-Jones et al., 2014).

Although no formal standards for the evaluation of use of theory in studies exist, there are a number of measures and conventions to test the model fit and utility of the statistical model. We report all goodness of fit tests which were presented by the primary study authors. The R-squared test (Cameron and Windmeijer, 1997) and the Hosmer and Lemeshow test (Hu and Bentler, 1999) are widely used for linear and logistic regression models. For these tests, the variability of the response data explained by the statistical model is provided as a proportion between 0% and 100%. The greater the proportion explained, the better the fit of the statistical model. Internal consistency of the statistical model can be indicated using Cronbach's alpha: a proportion less than 60% suggests an unacceptable level of internal consistency (Knapp, 1991). In confirmatory factor analysis, the fit of the statistical model can be assessed using CMIN/DF (chi-square divided by the df value) with a value close to one indicating a good fit. A ratio greater than two represents an inadequate fit (Kline, 1998, Bollen, 1989).

## Results

### Summary of relevant studies identified

**Fig. 1:**
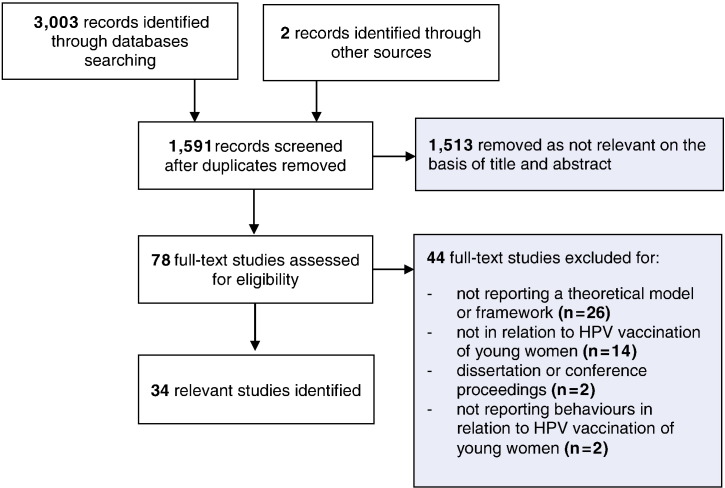
Flow diagram of study selection procedure.

3003 titles and abstracts were identified, of which 1591 were not duplicates. After screening titles and abstracts, 78 were considered to be potentially relevant; of these, 34 primary studies reported using at least one theoretical model or framework to explain behaviours in relation to HPV vaccination of young women (Fig. 1).

### Characteristics of studies

Thirty-one studies were identified which reported using at least one health behaviour theoretical model or framework (Bowyer et al., 2014, Spleen et al., 2012, Morales-Campos et al., 2013, Reiter et al., 2009, Dempsey et al., 2006, Natan et al., 2011, Reynolds and O'Connell, 2012, Roberto et al., 2011, Askelson et al., 2010a, Askelson et al., 2010b, Askelson et al., 2011a, Askelson et al., 2011b, Brawner et al., 2013, Gainforth et al., 2012a, Gainforth et al., 2012b, Hertweck et al., 2013, Kahn et al., 2005, Kahn et al., 2009, Ogilvie et al., 2007, Teitelman et al., 2011, Stretch et al., 2009, Fahy and Desmond, 2010, Brewer and Fazekas, 2007, Choi et al., 2013, D'Souza et al., 2011, Marlow et al., 2009, McRee et al., 2012, Rose et al., 2012, Thomas et al., 2013, Shafer et al., 2011, McSherry et al., 2012). The most widely reported were the theory of planned behaviour (Ajzen, 1985, Ajzen, 1991) (n of studies = 15, 44.1%) and the health belief model (Rosenstock et al., 1988, Hochbaum, 1958) (15, 44.1%). The theory of reasoned action (Fishbein and Ajzen, 1975, Ajzen and Fishbein, 1980) (n = 4, 11.8%), protection motivation theory (Rogers, 1975, Rogers, 1983) (2, 5.9%), prospect theory (Tversky et al., 1981) (2, 5.9%), and theoretical domains framework (Michie et al., 2005) (1, 2.9%) comprised the remainder. Three studies were identified which used sociocultural theories and frameworks: fundamental cause theory (Phelan and Link, 2005) (1, 2.9%), vaccine perceptions, accountability and adherence model (Katz et al., 2010) (1, 2.9%), and governmentality and disciplinary technologies of the self (Foucault, 1995) (1, 2.9%) (Table 1, Table 2). Five studies (14.7%) (Bowyer et al., 2014, Reiter et al., 2009, Hertweck et al., 2013, Teitelman et al., 2011, Polonijo and Carpiano, 2013) empirically tested applicability of the theoretical model in relation to the actual HPV vaccination status of young women.

The most frequently reported study design was cross-sectional questionnaire (n of studies = 18, 52.9%). Other study designs included: qualitative (7, 20.6%); development of an intervention (1, 2.9%); systematic review (1, 2.9%); experimental (2, 5.9%); prospective questionnaire (1, 2.9%); development of a questionnaire (1, 2.9%); interventions to increase HPV vaccine uptake (2, 5.9%); and mixed methods (1, 2.9%). Study participants included: parents (19, 55.9%); professionals involved in the HPV vaccination programme (8, 23.5%); young women (4, 11.8%), or; young women and their parents (4, 11.8%) (Table 1).

### Overview of primary study findings by theory or theoretical model

#### Theory of reasoned action and theory of planned behaviour

The theory of reasoned action (Fishbein and Ajzen, 1975, Ajzen and Fishbein, 1980) considers that behavioural intention is the best indicator of whether a specific behaviour is undertaken, and is influenced by a person's attitudes and subjective norms. The theory of planned behaviour (Ajzen, 1985, Ajzen, 1991) extended this to include perceived behavioural control.

Fifteen studies reported using the theory of planned behaviour (Bowyer et al., 2014, Roberto et al., 2011, Askelson et al., 2010a, Askelson et al., 2010b, Askelson et al., 2011a, Askelson et al., 2011b, Brawner et al., 2013, Gainforth et al., 2012a, Hertweck et al., 2013, Kahn et al., 2005, Kahn et al., 2009, Ogilvie et al., 2007, Teitelman et al., 2011, Stretch et al., 2009, Fahy and Desmond, 2010) and four studies used the theory of reasoned action (Dempsey et al., 2006, Natan et al., 2011, Reynolds and O'Connell, 2012, Roberto et al., 2011). Evidence was inconsistent as to which constructs influenced healthcare professionals' intentions to recommend vaccination (Roberto et al., 2011, Stretch et al., 2009, Kahn et al., 2005, Askelson et al., 2010b). Communication of sexually related information was also examined (Askelson et al., 2011b). Constructs identified to affect mothers' intentions to have their daughter vaccinated differed between studies (Reynolds and O'Connell, 2012, Askelson et al., 2010a, Askelson et al., 2011a, Gainforth et al., 2012a, Hertweck et al., 2013, Kahn et al., 2009, Ogilvie et al., 2007, Fahy and Desmond, 2010). Association between cultural and socioeconomic factors were observed in one study in Israel (Natan et al., 2011), but not in a study undertaken in Canada (Ogilvie et al., 2007). Mothers' intentions to communicate sexually related information with their daughter were also examined (Askelson et al., 2011a). In another study, the provision of written information was shown to be insufficient to change parental perceptions of vaccination of their daughters (Dempsey et al., 2006). Young women's intentions and behaviours were examined in three studies (Bowyer et al., 2014, Brawner et al., 2013, Teitelman et al., 2011). Relevant constructs varied in two of the studies (Brawner et al., 2013, Teitelman et al., 2011). In the UK setting, no constructs were found to be associated with uptake, but associations with lower uptake by ethnic group were found (Bowyer et al., 2014).

#### Health belief model

The health belief model (Rosenstock et al., 1988, Hochbaum, 1958) encompasses six main constructs to predict preventative behaviours: perceived susceptibility; perceived severity; perceived benefits; perceived barriers; self-efficacy; and call to action (Rosenstock et al., 1988, Hochbaum, 1958). Fifteen studies (Bowyer et al., 2014, Spleen et al., 2012, Morales-Campos et al., 2013, Reiter et al., 2009, Dempsey et al., 2006, Reynolds and O'Connell, 2012, Kahn et al., 2009, Brewer and Fazekas, 2007, Choi et al., 2013, D'Souza et al., 2011, Marlow et al., 2009, McRee et al., 2012, Rose et al., 2012, Thomas et al., 2013, Shafer et al., 2011) were identified as to which reported using the health belief model. In a systematic review of USA-based studies, the authors reported that parental acceptability of the HPV vaccine related to beliefs in effectiveness, susceptibility to HPV infection, and physician recommendation and barriers included cost and promotion of adolescent sexual behaviour (Brewer and Fazekas, 2007). One study found that parents' perceived barriers and harms of the HPV vaccine, and perceived likelihood of their daughter developing cervical cancer, were related to vaccination status of their daughter (Reiter et al., 2009). Korean school health teachers' intentions to recommend vaccination (Choi et al., 2013), parental intentions to have their daughter vaccinated against HPV (Reynolds and O'Connell, 2012, Kahn et al., 2009, Rose et al., 2012), and information seeking behaviour (McRee et al., 2012) were examined using the health belief model.

Further, the model was used in developing interventions to increase parents' intention to vaccinate their daughters (Spleen et al., 2012) and increase uptake (Dempsey et al., 2006, Brawner et al., 2013). Communication of messages targeting mothers of vaccine eligible young women (Shafer et al., 2011), a qualitative study examining Hispanic mothers' and daughters' perceptions of the HPV vaccine (Morales-Campos et al., 2013), and the Parental HPV Survey (Thomas et al., 2013) were also captured. Three studies reported using the health belief model to explain young women's behaviour in relation to the HPV vaccine (Bowyer et al., 2014, D'Souza et al., 2011, Marlow et al., 2009), but the influential domains were inconsistent.

#### Protection motivation theory

Protection motivation theory (Rogers, 1975, Rogers, 1983) predicts that the intention to protect depends upon four factors: perceived susceptibility; perceived severity; response efficacy: and perceived self-efficacy (Rogers, 1975, Rogers, 1983). Two studies used the protection motivation theory (Gainforth et al., 2012a, Gainforth et al., 2012b). Response efficacy, self-efficacy, and subjective norms in the Canadian school-based vaccination programme were identified to influence mothers' intentions to vaccinate their daughters (Gainforth et al., 2012a). Message framing did not influence Canadian parents' intentions to have their daughter vaccinated (Gainforth et al., 2012b).

#### Prospect theory

Prospect theory (Tversky et al., 1981) proposes that gains and losses are valued differently, which in turn can alter decision-making. ‘Gain frames’ highlight the benefits of complying with a recommended behaviour or avoidance of negative consequences. ‘Loss frames’ portray the negative consequences of noncompliance. In one study, no differences in effectiveness of ‘gain framed’ versus ‘loss framed’ messages to increase Irish parents' HPV vaccination intentions were observed (Fahy and Desmond, 2010). However, in another study in a USA setting, mothers were reported to respond more favourably to positive messages (Shafer et al., 2011).

#### Theoretical domains framework

The theoretical domains framework (Michie et al., 2005) integrated multiple behaviour change theories to include 12 domains: (i) knowledge; (ii) skills; (iii) social/professional role and identity; (iv) beliefs about capabilities; (v) beliefs about consequences; (vi) motivation and goals; (vii) memory, attention, and decision processes; (viii) environmental context and resources; (ix) social influences; (x) emotion regulation; (xi) behavioural regulation; and (xii) nature of the behaviour. In one study, all domains were identified to be related to Irish healthcare professionals behaviour, with the exception of the ‘memory, attention, and decision process’ construct (McSherry et al., 2012).

#### Fundamental cause theory

Fundamental cause theory (Phelan and Link, 2005) argues that health disparities persist because those with higher socioeconomic position have greater access to resources which can improve health. One study used fundamental cause theory to examine the potential impact of the USA HPV vaccination programme on future cervical cancer inequalities, and found unequal parental knowledge and receipt of a health professional recommendation contributed to disparities in uptake by ethnicity and socioeconomic status (Polonijo and Carpiano, 2013).

#### Governmentality and disciplinary technologies of the self

The term ‘governmentality’ was developed by Foucault in relation to how the power exercised by the state influences the way people conduct themselves (Foucault, 1995). One qualitative study found evidence of power relations through the aggressive marketing of the HPV vaccine, to Canadian healthcare professionals and parents, by pharmaceutical companies (Mishra and Graham, 2012).

#### Vaccine perceptions, accountability and adherence model

This (Katz et al., 2010) is a conceptual framework developed from the health belief model which incorporates wider structural, socio-cultural, and environmental factors and considers factors affecting completion separately to initiation. One qualitative study suggested that HPV vaccine uptake in South Africa has the potential to be influenced by the wider socio-cultural environment with high HIV endemicity, sexual violence, and poverty endemic poverty (Katz et al., 2013).

### Assessment of use of theoretical model or theory in primary studies

Overall, the majority (27 of 34) of studies presented the use of the theory or theoretical model consistently throughout the research process. It appeared that the theory guided and directed the various phases of the research process which could be tracked throughout the article (Bradbury-Jones et al., 2014). There were seven studies that appeared (from the information presented in the paper) to inconsistently use theory (Spleen et al., 2012, Morales-Campos et al., 2013, Dempsey et al., 2006, Stretch et al., 2009, Choi et al., 2013, Rose et al., 2012, Katz et al., 2013). One did not justify the selected theory within the manuscript (Rose et al., 2012). In two qualitative studies, the authors reported that the interview guide was developed using a theory but did not present study findings with reference to the theory (Morales-Campos et al., 2013, Stretch et al., 2009). Similarly, in a study reporting an intervention developed using the health belief model, the authors did not analyse the data using the health belief model (Spleen et al., 2012). Two studies did not explicitly refer to their theoretical framework in the discussion of their research findings (Dempsey et al., 2006, Katz et al., 2013), although one of these mentioned the limitations of a theoretical approach within the discussion (Dempsey et al., 2006) (Table 2). Restrictions imposed on authors, such as journal word limits and preferences, may have limited their ability to explicitly state their use of theory.

Internal consistency by grouping constructs was reported by 19 studies (Bowyer et al., 2014, Reiter et al., 2009, Dempsey et al., 2006, Natan et al., 2011, Reynolds and O'Connell, 2012, Askelson et al., 2010a, Askelson et al., 2010b, Askelson et al., 2011a, Askelson et al., 2011b, Gainforth et al., 2012a, Gainforth et al., 2012b, Kahn et al., 2005, Kahn et al., 2009, Ogilvie et al., 2007, Teitelman et al., 2011, Fahy and Desmond, 2010, Choi et al., 2013, Marlow et al., 2009, Thomas et al., 2013), of which ten studies (Bowyer et al., 2014, Reiter et al., 2009, Dempsey et al., 2006, Brawner et al., 2013, Ogilvie et al., 2007, Teitelman et al., 2011, Fahy and Desmond, 2010, Kahn et al., 2005, Askelson et al., 2010a, Askelson et al., 2010b) indicated lower internal consistency by some constructs (range: 20% to 65%). Lower explanatory power (< 70%) of theoretical models was identified within ten studies (Bowyer et al., 2014, Natan et al., 2011, Askelson et al., 2010a, Askelson et al., 2011a, Askelson et al., 2011b, Teitelman et al., 2011, Kahn et al., 2005, Choi et al., 2013, Marlow et al., 2009, Thomas et al., 2013) (range: 10% to 68%). Higher explanatory power (≥ 70%) was reported in seven studies (Reiter et al., 2009, Reynolds and O'Connell, 2012, Brawner et al., 2013, Gainforth et al., 2012a, Teitelman et al., 2011, Fahy and Desmond, 2010, Askelson et al., 2010b) (range: 70% to 96%). There were four quantitative studies which did not report testing the goodness of fit of the data of their theoretical model in the study (Spleen et al., 2012, McRee et al., 2012, Rose et al., 2012, Polonijo and Carpiano, 2013). This could either be as a result of selective non-reporting by the authors or that it had not been undertaken (Table 2).

## Discussion

The study aimed to provide an overview of the theoretical models or frameworks used to explain behaviours in relation to HPV vaccination of young women. Overall, 34 primary studies were identified which investigated a wide range of issues including: intentions to vaccinate or recommend vaccination against HPV; communication of information related to sexual transmission of HPV; interventions to increase acceptability; development of a questionnaire; power relationships; and explanation of health inequalities. The primary studies targeted a wide range of population groups, with parents predominating, in addition to healthcare professionals and young women themselves. Theory appeared to be consistently used by the primary studies throughout the research process to examine issues. The majority of quantitative studies that used behaviour change theory constructs gave an indication of the goodness of fit. However, some of the studies failed to report goodness of fit tests, or the statistical models presented and explained only a small proportion of the variability in the data.

Theoretical models related to individuals' health-related behaviour were predominantly used by the primary studies, of which the Theory of Reasoned Action, Theory of Planned Behaviour and the Health Belief Model were the most frequently reported. Behavioural theories and models are considered an important tool in effective behaviour change interventions and programmes (Craig et al., 2008, Campbell et al., 2000). Across a wide range of settings, relationships between internal constructs from the Theories of Planned Behaviour and Reasoned Action which measure individual motivational factors based on behavioural, normative and control beliefs were identified. Relationships between individuals' beliefs in relation to threat perception and behavioural evaluation, informed by the Health Belief Model, were also widely reported. These potentially modifiable beliefs can shape individual behaviour and can be targeted in order to bring about health-related behaviour changes.

The importance of the individual constructs as determinants of intentions and HPV vaccine-related behaviour varied by the population under consideration. For example, constructs of the Theory of Planned behaviour identified to be associated with Irish, American and Canadian mothers' intentions to vaccinate their daughters were inconsistent (Reynolds and O'Connell, 2012, Askelson et al., 2010a, Askelson et al., 2011a, Gainforth et al., 2012a, Hertweck et al., 2013, Kahn et al., 2009, Ogilvie et al., 2007, Fahy and Desmond, 2010). This highlights that, to be effective, individual-level behavioural interventions to increase HPV vaccination uptake may need to be adapted to the specific needs of the population under study. Similar to the findings presented in this study, a recent systematic review which use of theories of behaviour changes to prevent communicable diseases identified individual-level theories and models were most frequently used. However, less than half of the interventions which used theories based on individual-level behaviour were found to be effective (Angus et al., 2013).

The strength of an individualistic approach is the ability to highlight the complexity of factors which contribute to the behaviour of groups of individuals (Angus et al., 2013). However, we consider that there are a number of important shortfalls of the approaches undertaken by the studies in relation to developing interventions to increase uptake of HPV vaccination programmes.

Firstly, only five studies (Bowyer et al., 2014, Reiter et al., 2009, Hertweck et al., 2013, Teitelman et al., 2011, Polonijo and Carpiano, 2013) empirically tested the assumptions and applicability of the theoretical model in relation to the actual, or self-reported, vaccination status of young women. Consequently, there is little available evidence of the extent that individual determinants of behaviour contribute to uptake, or inequalities in uptake, of the HPV vaccination programme, and knowledge about how to change behaviour is currently limited. This may reflect greater challenges in obtaining young people's consent for research.

The studies infrequently reported wider determinants of health, such as social, economic, and environmental factors. These can either directly or indirectly affect an individual's ability or power to perform a specific behaviour. Although a number of psychological theoretical models or frameworks, for instance the health belief model, include factors relating to wider determinants (such as perceived costs) the focus is on individual perceptions and beliefs rather than structural constraints. Only two studies acknowledged this limitation in the discussion (Bowyer et al., 2014, Dempsey et al., 2006). Understanding wider determinants is important because interventions designed to prompt individual-level behaviour change, by focusing on a person's perceptions or attitudes, risk being ineffective if the other factors governing uptake are not simultaneously addressed such as policies and procedures influencing access to healthcare facilities, and the availability or affordability of the vaccine.

Health psychological theoretical models or frameworks are biased towards rationalistic, volitional human behaviour by which an individual decides on, and commits to, a particular behaviour in a logical manner in order to maximise health benefits (Taylor et al., 2007). This ignores that behaviour is largely determined by combinations of circumstantial reality and individuals' habitual, emotional and unconscious reactions to the external world (Taylor et al., 2007). In addition, the theoretical models or frameworks fail to account for external influences that can alter behaviour between decision-making and performing the behaviour.

Finally, none of the studies attempted to understand how interactions between different groups of people influence uptake of the HPV vaccine. This is important because HPV decision-making involves interactions between different combinations of policy makers, healthcare professionals, and community or religious leaders, as well as parents and young women. This did not appear to be acknowledged in the studies (Batista Ferrer et al., 2014).

### Incorporating wider determinants of health in relation to HPV vaccine uptake

The limitations of using theories of individual behaviour to explain HPV vaccination uptake can be counteracted using a theoretical model or framework which encompasses intra- and inter-personal behaviour and also acknowledges the wider determinants of behaviour. Ecological frameworks, such as the socio-ecological (McLeroy et al., 1988, Reifsnider et al., 2005), structural–ecological (Cohen et al., 2000), and the social-ecology (Stokols, 1992) models are of particular relevance. Ecological frameworks may provide more comprehensive frameworks for understanding the multiple and interacting determinants of health behaviours which may operate at several or all of following levels: public policy; community; organisational; interpersonal; and intrapersonal. Importantly, an ecological framework assists with the identification of appropriate levels at which to target interventions informed by relevant theories.

A qualitative systematic review and evidence synthesis illustrated how a young woman's access to the HPV vaccine is shaped by decisions at different levels of the socio-ecological model (Batista Ferrer et al., 2014) including: the policy context in relation to costs and accessibility; social norms and values of sexual activity and vaccine beliefs; the views and actions of healthcare professionals; and parental consent procedures (Batista Ferrer et al., 2014). This supports how an intervention aimed at individual-level changes to behaviour is unlikely to be successful if other barriers are not simultaneously addressed.

## Conclusion

Currently, it is difficult to draw firm conclusions about the contribution of theory promoting HPV vaccination uptake and addressing inequalities due to a wide variety of approaches and inconsistent findings from any single theory. The use of theoretical models and frameworks is heavily weighted towards intra- and inter-personal factors that affect individuals' intentions. We suggest that a more comprehensive approach, which also accounts for the broader social, cultural and political context, is required. Given the small number of studies that examined ecological frameworks, combined with the importance of these factors in predicting behaviour, more research is required to examine whether such frameworks can assist in developing interventions which increase uptake of HPV vaccination programmes.

## Conflicts of interest statement

HB-F, MH and SA have no conflicts of interest to declare. CT received consultancy payment from GSK for a critical review of a health economic model of meningococcal ACWY vaccine.

## Figures and Tables

**Table 1:** Characteristics of studies about HPV vaccination of young women reporting using health behaviour theoretical models.

Authors	Publication year	Study aim	Study design	Participants
Askelson et al. (2010b)	2010	To assess factors related to physicians' intentions to vaccinate patients against human papillomavirus	Cross-sectional questionnaire	Physicians
Askelson et al. (2010a)	2010	To assess mothers' intentions to vaccinate their daughters against human papillomavirus	Cross-sectional questionnaire	Mothers
Askelson et al. (2011b)	2011	To assess intentions for physicians to talk about sexually related information in relation to HPV vaccine with young women	Cross-sectional questionnaire	Physicians
Askelson et al. (2011a)	2011	To examine mothers' intentions to talk about sexually related information in relation to HPV vaccine with young women	Cross-sectional questionnaire	Mothers
Bowyer et al. (2014)	2013	To examine psychosocial predictors of HPV vaccine uptake and the association between vaccine intention and uptake 1 year later	Prospective questionnaire	Adolescent girls
Brawner et al. (2013)	2013	To describe the development of two skills-based intervention curricula aimed to increase HPV prevention and vaccination	Intervention development	Adolescent females and parents/guardians
Brewer and Fazekas (2007)	2007	To systematically review studies of HPV-related beliefs and HPV vaccine acceptability	Systematic review	Not applicable
Choi et al. (2013)	2013	To determine the predictors of school health teachers' intention to recommend the HPV vaccine to students	Cross-sectional online survey	School health teachers
Dempsey et al. (2006)	2006	To identify independent predictors associated with HPV vaccine acceptability by parents	Intervention study	Parents
D'Souza et al. (2011)	2011	To shed light on the uptake of the HPV immunisation	Qualitative study (focus groups)	Young women
Fahy and Desmond (2010)	2010	To examine the effect of message framing on mother's intentions to obtain HPV vaccination for their teenage daughters	Experimental	Mothers
Gainforth et al. (2012a)	2012	To understand the determinants of vaccination intentions of mothers of young women	Cross-sectional questionnaire	Parents
Gainforth et al. (2012b)	2012	To investigate the effect of framed messages on parents' intentions to have their children vaccinated against HPV	Quasi experimental design	Parents
Hertweck et al. (2013)	2013	To understand the predictors of a mother's decision behaviour to vaccinate her daughter	Cross-sectional questionnaire	Mothers
Kahn et al. (2005)	2005	To examine paediatrician characteristics and attitudes associated with intention to recommend two hypothetical HPV vaccines	Cross-sectional questionnaire	Paediatricians
Kahn et al. (2009)	2009	To examine mothers' intention to vaccinate their daughters against HPV	Cross-sectional questionnaire	Mothers
Katz et al. (2013)	2013	To examine both adolescents' and caregivers' views of HPV vaccination to understand factors influencing uptake	Qualitative	Adolescents & their caregivers
Marlow et al. (2009)	2009	To assess acceptability of HPV vaccination amongst female adolescents	Cross-sectional questionnaire	Young women
McRee et al. (2012)	2012	To examine associations between parents' Internet information-seeking and their knowledge, attitudes and beliefs about HPV vaccine	Cross-sectional telephone survey	Parents
McSherry et al. (2012)	2012	To identify HPV-related clinical behaviours	Qualitative	Primary care practitioners
Mishra and Graham (2012)	2012	To examine whether HPV vaccination is conjoined with the power relationships over women's bodies	Qualitative	Vaccine scientists and healthcare providers
Morales-Campos et al. (2013)	2012	To assess Hispanic mothers' and girls' perceptions about cervical cancer, HPV and the HPV vaccine	Qualitative	Mothers and daughters
Natan et al. (2011)	2011	To examine whether the model based on the Theory of Reasoned Action succeeds in predicting mothers' intention to vaccinate their daughters against HPV	Cross-sectional questionnaire	Mothers
Ogilvie et al. (2007)	2007	To determine intentions regarding HPV vaccination amongst Canadian parents and factors that predict parental intention	Cross-sectional telephone survey	Parents
Polonijo and Carpiano (2013)	2013	To examine distinct stages in which disparities of uptake of the HPV vaccine may arise	Cross-sectional telephone survey	Parents
Reiter et al. (2009)	2009	To identify modifiable correlates of HPV initiation amongst adolescent girls in high risk communities	Cross-sectional telephone survey	Parents
Reynolds and O'Connell (2012)	2012	To test a model that predicts intention to vaccinate and vaccine status	Cross-sectional online survey	Parents
Roberto et al. (2011)	2011	To examine the ability of the theory of reasoned action and the theory of planned behaviour to predict whether or not paediatricians encourage parents to get their adolescent daughters vaccinated against HPV	Cross-sectional questionnaire	Paediatricians
Rose et al. (2012)	2012	To identify factors predictive of parents' intent to have their daughters' receive the HPV vaccine	Cross-sectional questionnaire	Parents
Shafer et al. (2011)	2011	To develop HPV vaccine messages for a campaign targeting racially diverse mothers of nonvaccinated 11–12-year-old girls	Intervention development: Qualitative	Mothers
Spleen et al. (2012)	2012	To assess change in HPV related knowledge and parental intent to vaccinate daughters	Intervention	Parents
Stretch et al. (2009)	2009	To seek the views of school nurses on vaccinating girls who did not have parental consent for HPV vaccination	Qualitative	School nurses
Teitelman et al. (2011)	2011	To identify common beliefs about HPV vaccine initiation	Mixed methods (questionnaire and interviews)	Young women
Thomas et al. (2013)	2013	To develop a survey to assess parents regarding their HPV knowledge, attitudes, and intent to vaccinate	Questionnaire development	Parents/caregivers

**Table 2:** Studies use of health behaviour and socio-cultural theories and frameworks.

Authors	Theoretical model	Analytical approach	Reporting of use of theoretical framework	Appropriateness of model	Overall study findings
Askelson et al. (2010b)	Theory of planned behaviour	Structural equation modelling	Consistently reported	Internal consistency: 31% to 99%; Comparative fit index: 96%	Associations by subjective norms (p < 0.05) and perceived behavioural control (p < 0.05)
Askelson et al. (2010a)	Theory of planned behaviour	Confirmatory factor & linear regression model	Consistently reported	Internal consistency: 38% to 96%; Linear regression: accounts for 66% of variance	Overall intentions not high; Associations by attitudes (p > 0.01) and subjective norms (p > 0.05)
Askelson et al. (2011b)	Theory of planned behaviour	Principal components & linear regression model	Consistently reported	Internal consistency: 73% to 91%; Linear regression: accounts for 52% of variance	Associations by attitudes (p < 0.05), subjective norms (p < 0.001), and perceived behavioural control (p < 0.05)
Askelson et al. (2011a)	Theory of planned behaviour	Principal components & linear regression model	Consistently reported	Internal consistency: 80% to 83%; Linear regression: accounts for 37% of variance	Associations by attitudes (p < 0.01), subjective norms (p < 0.001), and age of vaccination (p < 0.05)
Bowyer et al. (2014)	Health belief model & Theory of planned behaviour	Complex samples logistic regression analysis	Consistently reported	Internal consistency: 69% to 97%; Accounts for 14% to 19% of variance	Associations by ethnicity (p = 0.03)
Brawner et al. (2013)	Theory of planned behaviour	Descriptive analyses	Consistently reported	Accounts for 29% to 79% of the variance	Associations with adolescents' attitudes, normative beliefs, and perceived behavioural control (p-values not provided)
Brewer and Fazekas (2007)	Health belief model	Narrative review	Consistently reported	Not applicable	Acceptability related to beliefs in effectiveness, susceptibility to HPV infection, and physician recommendation; Barriers: cost and promotion of adolescent sexual behaviour
Choi et al. (2013)	Health belief model	Stepwise multiple regression analysis	Inconsistently reported: Discussion did not explicitly relate to model	Internal consistency: 71% to 92%; Accounts for 15% of variance	Associations by perceived benefits (p = 0.006), education needs (p = 0.013), and age of school teachers (p = 0.04)
Dempsey et al. (2006)	Health belief model & Theory of reasoned action	Multivariable linear regression analysis	Inconsistently reported: Discussion did not explicitly relate to model; Authors discussed limitations of using theoretical frameworks	Internal consistency: 20% to 83%	Parents who received the HPV information sheet had higher mean scores. Benefits of HPV vaccines (p < 0.001), peer group influences (p < 0.004), physician recommendation (p < 0.001), perceived susceptibility (p < .001), personal experience (p = 0.042); Perceived barriers: discomfort or danger when receiving immunisations (p < 0.001)
D'Souza et al. (2011)	Health belief model	Qualitative (not explicit)	Consistently reported	Not applicable	Awareness about HPV vaccine was low; Free cost of vaccine encouraged vaccination; Social capital and trust can improve communication strategies
Fahy and Desmond (2010)	Theory of planned behaviour & Prospect theory	Multivariate analysis of variance	Consistently reported	Internal consistency: 47% to 80%; Linear model: accounts for 70% of variance	No effect by message frame (p = 0.40); Associations of intentions with positive attitudes towards HPV vaccination (< 0.001), influence of peer groups (p = 0.008) and recommendations by healthcare professionals (p = 0.04)
Gainforth et al. (2012a)	Protection motivation theory & Theory of planned behaviour	Principal components and linear regression analysis	Consistently reported	Internal consistency: 84% to 95%; Linear model: accounts for 56% of variance	Associations by response efficacy (p < 0.001), self-efficacy (p < 0.01) and subjective norms (p < 0.001)
Gainforth et al. (2012b)	Protection motivation theory	ANCOVA	Consistently reported	Internal consistency: All greater than 84%	Message framing had no effect on intentions to vaccinate or for parents to talk to healthcare professionals
Hertweck et al. (2013)	Theory of planned behaviour	Structural equation model	Consistently reported	CMIN/DF = 0.87	Associations by attitude (p < 0.001), subjective norms (p < 0.001), and perceived behavioural control (p < 0.001)
Kahn et al. (2005)	Theory of planned behaviour	Principal components and multivariable linear regression analysis	Consistently reported	Internal consistency: 65% to 88%; Regression model: accounts for 9% of variance	Higher estimate of the percentage of sexually active adolescents in one's practice (p = 0.002), number of young adolescents seen weekly (p = 0.015), higher HPV knowledge (p = 0.015), likelihood of following the recommendations of important individuals and organisations regarding immunisation (p = 0.001), and fewer perceived barriers to immunisation (p = 0.001)
Kahn et al. (2009)	Health belief model & Theory of planned behaviour	Multivariable logistic regression analysis	Consistently reported	Internal consistency: 75%; Variance not reported	Beliefs about Pap tests and HPV vaccine (p-values not reported)
Katz et al. (2013)	Vaccine perceptions, accountability, and adherence model	Inductive approach based upon grounded theory	Inconsistently reported: Discussion did not explicitly relate to model	Not applicable	Uptake driven by socio-cultural environment with high HIV endemicity, sexual violence, poverty, and female headed households
Marlow et al. (2009)	Health belief model	Factor loading and multivariable logistic regression analysis	Consistently reported	Internal consistency: 78% to 87%; Logistic regression model: accounts for 19% of variance	Religion, perceived susceptibility, benefits and barriers were associated (all p < 0.01)
McRee et al. (2012)	Health belief model	Multivariable logistic regression analysis	Consistently reported	Not reported	Associations by perceived likelihood of HPV (p < 0.05), uncertainty (p < 0.001) and perceived harms of HPV vaccine (p < 0.001). In addition to anticipated regret (p < 0.05) and higher HPV knowledge (p < 0.001)
McSherry et al. (2012)	Theoretical domains framework	Content analysis	Consistently reported	Not applicable	Beliefs about consequences, social influences, knowledge and environmental context, and resources most influential
Mishra and Graham (2012)	Governmentality and disciplinary technologies of the self	Thematic analysis	Consistently reported	Not applicable	Evidence of power relationships and gender issues in relation to the introduction of the HPV vaccination programme
Morales-Campos et al. (2013)	Health belief model	Grounded theory	Inconsistently reported: Informed interview guide but not clearly referred to in discussion	Not applicable	Gaps in knowledge, fears and concerns about the vaccine, sociocultural communication practices, and decision-making
Natan et al. (2011)	Theory of reasoned action	Factor loading and multivariable linear regression analysis	Consistently reported	Internal consistency 78% to 83%. Linear model accounts for 43% of variance	Associations by behavioural beliefs (p < 0.03), normative beliefs (p < 0.001), knowledge (p = 0.04), and religiosity (p < 0.001)
Ogilvie et al. (2007)	Theory of planned behaviour	Factor loading and backward logistic regression analysis	Consistently reported	Internal consistency 30% to 90%; Variance not reported	Associations with positive attitudes towards vaccines, subjective norms, limited influence on sexual behaviour and experience of cervical cancer; Parental age and region of residence also identified; p-values not provided
Polonijo and Carpiano (2013)	Fundamental cause theory	Logistic regression analysis	Consistently reported	Not reported	Unequal parental knowledge and receipt of a health professional recommendation contribute to disparities in uptake
Reiter et al. (2009)	Health belief model	Multivariate logistic regression model	Consistently reported	Internal consistency: 64% to 70%;	Perceived harms of HPV vaccine (p < 0.01), perceived barriers to vaccination (p < 0.01), and perceived likelihood of cervical cancer (p < 0.01)
Reynolds and O'Connell (2012)	Health belief model & Theory of reasoned action	Principal components factor analysis and logistic regression	Consistently reported	Internal consistency: 73% to 95%; Hosmer and: Lemeshow test: predicts 84%	Associations by social norms (p = 0.001), attitudes (p < 0.001) and age of child (p < 0.001)
Roberto et al. (2011)	Theory of reasoned action & Theory of planned behaviour	Stepwise regression	Consistently reported	Not reported	Positive attitudes (p < 0.01), subjective norms (p < 0.01), and perceived behavioural control (p < 0.01)
Rose et al. (2012)	Health belief model	Backwards selection regression model	Inconsistently used: Use of model not justified in text	Not reported	Associations with fewer negative views on vaccination (p<0.01), adequate information (p<0.01), perceiving HPV infection and cervical cancer as serious and likely to occur (p<0.01), importance of effectiveness and safety (p<0.01), and parental education (p=0.02)
Shafer et al. (2011)	Health belief model & Prospect theory	Constant comparison method	Consistently reported	Not applicable	Mothers wanted to protect their daughter from harm and preferred messages in relation to prevention of cervical cancer
Spleen et al. (2012)	Health belief model	Pre- and post-intervention analysis	Inconsistently reported: Intervention developed guided by the health belief model; Results and discussion to not refer to model	Not applicable	HPV-related knowledge (p < 0.0001) and intent to vaccinate (p < 0.002) increased post intervention
Stretch et al. (2009)	Theory of planned behaviour	Thematic analysis	Inconsistently reported: Informed interview guide but not clearly referred to in discussion	Not applicable	Positive beliefs in relation to vaccines and benefits of preventing HPV acquisition, healthcare professionals hesitant to assess ‘Gillick competency’ in the absence of parental consent
Teitelman et al. (2011)	Theory of planned behaviour	Factor loading and multivariable general linear modelling; Content analysis	Consistently reported	Internal variance: 81% to 96%; Linear model: Accounts for 49% to 77% of the variance	Associations with attitudes, norms, perceived control, and tobacco use (all p < 0.001)
Thomas et al. (2013)	Health belief model	Classical item analysis and exploratory factor analysis	Consistently reported	Internal variance: 96%; Factor analysis accounts for 62% to 68% of variance.	Not reported
